# Structural and functional analysis of the solute-binding protein UspC from *Mycobacterium tuberculosis* that is specific for amino sugars

**DOI:** 10.1098/rsob.160105

**Published:** 2016-06-22

**Authors:** Elizabeth Fullam, Ivan Prokes, Klaus Fütterer, Gurdyal S. Besra

**Affiliations:** 1 School of Life Sciences, University of Warwick, Coventry CV4 7AL, UK; 2 School of Biosciences, University of Birmingham, Edgbaston, Birmingham B15 2TT, UK; 3 Department of Chemistry, University of Warwick, Coventry CV4 7AL, UK

**Keywords:** amino sugars, X-ray crystallography, *Mycobacterium tuberculosis*, ATP-binding cassette transporters, nutrient acquisition, peptidoglycan

## Abstract

*Mycobacterium tuberculosis* (*Mtb*), the aetiological agent of tuberculosis, has evolved to scavenge nutrients from the confined environment of host macrophages with mycobacterial ATP-binding cassette (ABC) transporters playing a key role in nutrient acquisition. *Mtb*-UspC (Rv2318) is the solute-binding protein of the essential transporter UspABC, one of four *Mtb* ABC transporters implicated by homology in sugar acquisition. Herein, we report the structural and functional characterization of *Mtb*-UspC. The 1.5 Å resolution structure of UspC reveals a two subdomain architecture that forms a highly acidic carbohydrate-substrate binding cleft. This has allowed a distinct preference of *Mtb*-UspC for amino sugars as determined by thermal shift analysis and solution saturation transfer difference-NMR. Taken together our data support the functional assignment of UspABC as an amino-sugar transporter. Given the limited availability of carbohydrates within the phagosomal environmental niche during *Mtb* intracellular infection, our studies suggest that UspABC enables *Mtb* to optimize the use of scarce nutrients during intracellular infection, linking essentiality of this protein to a potential role in recycling components of cell-wall peptidoglycan.

## 1. Introduction

*Mycobacterium tuberculosis* (*Mtb*) is a major human pathogen and is the causative agent of tuberculosis (TB). TB remains a major global health threat and is the leading cause of mortality worldwide from a single infectious agent, with an excess of nine million new cases of TB each year claiming the lives of 1.5 million people annually [1]. While TB can be treated, the regimen extends over six to nine months. Premature termination of therapy in combination with a static pool of anti-tubercular drugs are among the major factors in causing the emergence of drug-resistant strains, which now includes extensively drug-resistant and untreatable forms of the disease [2,3]. Clearly, there is an urgent need to address this global health problem.

*Mtb* is a facultative intracellular organism able to evade the host immune response and survive within phagosomes for decades. Within this environment, *Mtb* has restricted access to nutrients, and mechanisms of nutrient supply during intracellular infection are poorly understood [4]. A growing body of evidence suggests that *Mtb* uses host lipids as the main carbon and energy source, reflected by, firstly, an over-representation of genes in the *Mtb* genome that encode enzymes of fatty acid metabolism [5] and, secondly, upregulation of such genes during macrophage infection [6]. Recent studies point to host lipid cholesterol as a major carbon source used by *Mtb* during infection. However, blocking cholesterol uptake and metabolism only partially attenuates *Mtb* virulence, suggesting that other yet to be identified carbon sources also have an important role to play [7,8].

It is generally assumed that access of *Mtb* to host sugars is particularly limiting. Bioinformatics analysis of the genome sequence of *Mtb* has led to the identification of a number of transporter systems, four of which have been annotated as carbohydrate importers of the ATP-binding cassette (ABC) superfamily [9–11]. Genome-wide saturation transposon mutagenesis studies by *Himar1* suggest a role for these systems in the virulence of *Mtb* [6,12]. Up until recently the substrates for these *Mtb* carbohydrate importers have remained elusive. However, it has since been demonstrated that the LpqY-SugABC transporter system is specific for the uptake of trehalose, which is recycled from the cell-wall glycolipid trehalose monomycolate [13,14]. Importantly, the LpqY-SugABC importer has been demonstrated to be essential for the virulence of *Mtb in vivo* [13]. Similarly, the solute-binding protein UgpB of the UgpAEBC transport system has been implicated in the recognition of *sn-*glycero-3-phosphocholine, a glycolipid that is upregulated during *Mtb* infection of guinea pigs [15,16]. Given the small number of carbohydrate import systems in *Mtb* (five) compared to, for instance, the soil-dwelling *Mycobacterium smegmatis* (28 ABC transporters) [10] it is plausible that this discrete set of transporters in *Mtb* is the result of adaptation to a very limited set of carbohydrates available in the host environment. Functional roles and substrate specificities of the remaining putative carbohydrate transporters, which include the SugI permease, Rv2038-Rv2041 ABC transporter and the UgpABCE ABC transporter, are not yet known.

UspC from *Mtb* is a 441-amino acid protein that has been previously identified in bioinformatics analyses as a putative active importer of carbohydrates across the inner-membrane of the *Mtb* cell wall [9,17]. The *uspC* gene forms part of a putative three-gene operon, *uspABC*, of which *uspA* and *uspB* encode the membrane-spanning subunits of the transporter, while *uspC* is a homologue of ABC transporter-linked solute-binding proteins. The operon lacks an obvious candidate for encoding the nucleotide-binding domain (NBD), which remains to be identified. It is probable that the UspABC transporter shares the NBD with another mycobacterial ABC transporter [9], which is not unusual among bacterial ABC transporters [18]. The *Mtb* UspABC ABC transporter has been demonstrated to be essential for growth *in vitro* [19] and is conserved in *Mycobacterium leprae*, an obligate pathogen that has undergone massive gene decay [20], resulting in a set of genes that are considered core for facilitating intracellular survival in humans. Conservation of *uspABC* in the *M. leprae* genome underscores the notion that it carries an indispensable function and is highly conserved across mycobacterial genomes (electronic supplementary material, figure S1 and table S1).

Similar to other substrate binding domains of Gram-positive ABC transporters, UspC is predicted to have an *N-*terminal membrane-associated anchor, comprising residues 7–29 (THMM server [21]), which does not appear to include a known signal peptidase cleavage site (SignalP [22]). Very little is known about the function and substrate(s) of UspC and the associated transporter system. Here, we report structural and biochemical evidence which demonstrates that UspC is able to selectively bind amino sugars, suggesting that in *Mtb* the UspABC ABC transporter may have a key function in the assimilation of amino sugars and hence have a role in optimizing the use of scarce nutrients available during intracellular infection.

## 2. Results

### 2.1. Production of N-terminally truncated UspC from *Mycobacterium tuberculosis*

The amino acid sequence of UspC includes an N-terminal 31-residue segment, of which residues 7–31 are predicted to form a trans-membrane anchor-helix, which negatively affected solubility of the full-length recombinant protein. Therefore, we generated an N-terminally truncated *Mtb*-*uspC* mutant, encoding residues 31–441, by PCR amplification and cloning this gene fragment into a pET-family plasmid containing either N*-*terminal or C*-*terminal hexa-histidine affinity tags. The expression of N-terminally truncated UspC (UspC_Nt_) in *Escherichia coli* resulted in 20 mg l^−1^ of soluble protein that could be purified to apparent homogeneity using Ni^2+^-affinity and anion exchange chromatography (electronic supplementary material, figure S2).

### 2.2. Crystal structure of *Mycobacterium tuberculosis* UspC_Nt_

**Figure 1.**
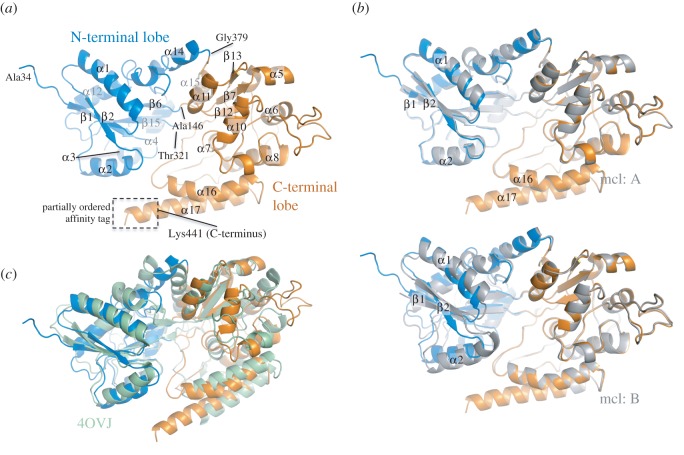
Overview of the structure of UspC_Nt_ and comparison with structural neighbour. (*a*) Ribbon representation of the structure of UspC_Nt_ (tetragonal crystal form) and identification of secondary structure elements. The partially ordered His6-affinity tag forms an extension to the C-terminal helix α17. (*b*) Superposition of the tetragonal structure with molecules A and B of the monoclinic crystal form. (*c*) Superposition of UspCNt with the closest structural neighbour according to secondary structure matching using PDBeFold [24]. The structural homologue is solute-binding protein family 1 from *Alicyclobacillus acidocaldarius* subsp. *acidocaldarius* DSM 446 (PDB entry 4OVJ).

UspC_Nt_ readily formed crystals in vapour diffusion experiments using a commercial sparse matrix screen (see Material and methods). Phases were determined by single-wavelength anomalous diffraction data to 2.6 Å (electronic supplementary material, figure S3), exploiting the anomalous signal from bound iodine ions. The structural model was refined against a native dataset (*apo* tetragonal, table 1) to a resolution of 1.5 Å (figure 1). The UspC_Nt_ structure determined represents the ligand-free form and the model comprises residues 34–441, plus six additional residues of the partially ordered C-terminal affinity tag (figure 1*a*). The fold of UspC_Nt_ follows the architecture of periplasmic binding proteins for bacterial ABC transporters, consisting of two subdomains or lobes that enclose the putative carbohydrate-binding cleft in the centre of the molecule. Both subdomains consist of two sequence segments, residues 34–146 and 321–379 for the N-terminal lobe, and residues 147–320 and 380–440 for the C-terminal lobe, respectively. The subdomains are joined by a central flexible hinge-linker that is localized around residues Asp145, Thr321 and Gly379 (figure 1*a*). The fold of the N-terminal subdomain is characterized by a central, mixed β-sheet (β1, β2, β6 and β15), flanked by α-helices on either face of the sheet. The C-terminal lobe is predominantly α-helical, with a small three-stranded β-sheet (β7, β12 and β13) that is surrounded by a cluster of helices (figure 1*a*).

**Table 1.** X-ray diffraction data and refinement statistics.

X-ray diffraction data			
crystal	*apo* tetragonal	*apo* monoclinic	iodine derivative
beamline	Diamond I04-1	Diamond I04	rotating anode
wavelength (Å)	0.9173	0.9795	1.5418
space group	*P*4_1_	*P*2_1_	*P*4_1_
cell parameters *a,b,c* (Å)	87.85, 87.85, 52.94	87.0, 52.4, 87.9, *β* = 90.9°	87.9, 87.9, 52.95
molecules in ASU	1	2	1
resolution (Å)	45.3–1.50	87.9–2.41	27.8–2.6
high-resolution shell (Å)	1.54–1.50	2.47–2.41	2.74–2.60
*R*_merge_ (%)^a^	10.6 (80.8)	11.3 (66.4)	9.2 (20.9)
total, unique reflections	437 432, 64 354	224 325, 30 838	1147489, 12662
*I*/σ(*I*)^a^	13.8 (3.2)	14.3 (2.3)	80.1 (33.7)
completeness (%)^a^	99.4 (99.0)	99.6 (95.2)	99.8 (99.3)
multiplicity^a^	6.8 (6.9)	7.3 (6.0)	90.6 (72.1)
anomalous completeness^a^			99.8 (99.0)
anomalous multiplicity^a^			43.4 (33.6)
ShelxC-<d″/sig>			3.8–1.1
FOM^b^ (27.8–2.6 Å)			0.359
**refinement**			
resolution range (Å)	45.3–1.50	87.9–2.41	
unique reflections	61 120	29 276	
*R*_cryst_, *R*_free_ (%)	16.2, 18.9	20.3, 26.1	
no. of non-hydrogen atoms	3620	5930	
protein, solvent	3131, 489	5878, 52	
r.m.s.d. bonds, angles (Å, °)	0.014, 1.59	0.015, 1.7	
Wilson B-factor (Å^b^)	12.3	38.9	
average—all atoms (Å^b^)	14.4	34.7	
protein, solvent (Å^b^)	12.4, 27.0	34.8, 24.5	
r.m.s.d. B-factors (Å^b^)	0.7	0.9	
Ramachandran plot^c^			
favoured region (%)	99	96.5	
allowed regions (%)	1	3.25	
disallowed (%)	0	0.25	

^a^Numbers in parentheses refer to the last resolution shell.

^b^FOM calculated after phasing, prior to density modification.

^c^Ramachandran plot statistics were calculated using Molprobity [23].

UspC_Nt_ crystallized in two different crystal lattices (table 1). The tetragonal crystal form (space group *P*4_1_) contained one copy of UspC_Nt_ in the crystallographic asymmetric unit (ASU), whereas the monoclinic crystal form (space group *P*2_1_) contained two copies. Molecule A of the monoclinic crystal form matches almost exactly the overall structure of the tetragonal crystal form (figure 1*b*, r.m.s.d. 0.4 Å for 400 aligned Cα positions), while molecule B shows a re-orientation of the N-terminal domain (figure 1*b*, r.m.s.d. 0.89 Å, 300 aligned Cα positions). The conformational flexibility of UspC_Nt_ revealed by the comparison of the monoclinic to the tetragonal crystal form highlights the potential for structural plasticity between the two domains, which may be functionally significant in ligand binding and is comparable to previously reported carbohydrate-binding domains of ABC transporters, which undergo an opening/closing motion upon ligand binding, exemplified by the structures of GacH (electronic supplementary material, figure S4) [18]. Comparison of molecule B of the monoclinic crystal form of UspC_Nt_ with the tetragonal structure demonstrates that UspC possesses the capacity to undergo a similar closing motion (figure 1*b*). Analysis of the packing interfaces of the monoclinic crystal form of UspC_Nt_, using the PISA server (http://www.ebi.ac.uk/msd-srv/prot_int/pistart.html [25]), does not suggest self-assembly of UspC_Nt_ into dimers or higher oligomers, in line with a gel filtration experiment where UspC_Nt_ (44 kDa) eluted between the 29 and 66 kDa calibration markers (electronic supplementary material, figure S5).

### 2.3. Comparison with other sugar solute-binding proteins of ATP-binding cassette transporters

The closest structural neighbour of UspC according to secondary structure matching (PDBeFold [24]) is the extracellular solute-binding protein from *Alicylclobacillus acidocaldarius* subsp. *acidocaldarius* DSM446 (PDB entry 4ovj, listed as ‘to be published’, no function assigned), aligning with an r.m.s.d. of 2.91 Å for 352 aligned residues (sequence identity 18%, figure 1*c*). The functional relationship of UspC with solute ABC transporters is further underscored by the alignment with the solute binder of the *E. coli* maltose transporter complex (PDB entry 3puw [26]), which appears as the second highest hit in the search of structural neighbours (r.m.s.d. 2.78 Å, 326 aligned Cα atoms, 18% sequence identity, electronic supplementary material, figure S6a). Furthermore, the recently determined structure of the *Mtb* solute-binding protein UgpB (PDB entry 4MFI [15]) also aligns closely with UspC (r.m.s.d. 2.95 Å, 326 aligned Cα atoms, sequence identity 17.8%, electronic supplementary material, figure S6b). UgpB is part of the UgpABCE transporter system, which has been implicated in the uptake of *sn*-glycero-3-phosphochline [15]. Thus, the structural comparison with functionally characterized ABC transporters supports the assignment of UspC as a component of an ABC transporter system.

### 2.4. The putative ligand-binding cleft of UspC_Nt_

**Figure 2.**
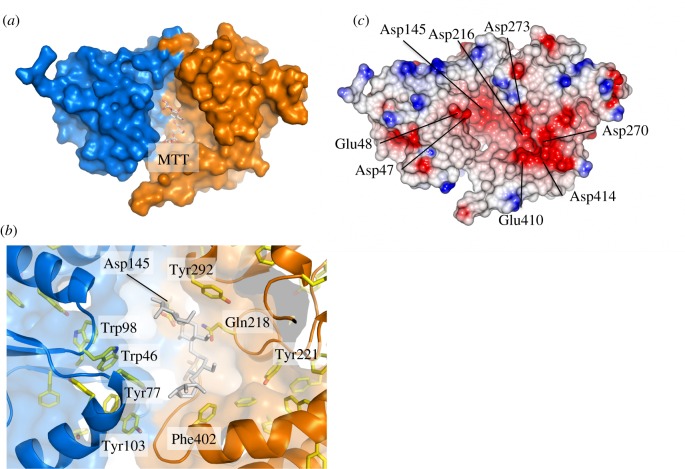
Putative substrate binding cleft of UspC. (*a*) Surface representation of UspC_Nt_ illustrating the putative ligand-binding cleft with N- and C-terminal lobes shown in blue and orange, respectively. Maltotetraose (MTT) was placed according to the secondary structure-matched superposition of UspC_Nt_ with MTT-bound GacH (PDB entry 3K00 [18]). (*b*) Close-up view of the putative substrate binding cleft, highlighting aromatic side chains with significant exposure to solvent and the residue cluster of Asp145, Gln218 and Tyr292 in the centre of the cleft, which were subjected to site-directed mutagenesis. Sticks in grey show the position of MTT as derived from superposition with MTT-bound GacH. (*c*) Electrostatic surface diagram of UspC_Nt_ generated in CCP4MG [27] shown in an orientation identical to panel (*a*).

The molecular surface of UspC_Nt_ shows a prominent cleft between the two subdomains (figure 2*a*), which is characteristic for periplasmic substrate binding proteins of ABC transporters. Apart from the structural similarity to functionally characterized solute-binding proteins, several structural features of the UspC_Nt_ inter-lobe cleft suggest a functional carbohydrate substrate binding unit of the UspABC transporter system. The cleft is lined by several aromatic side chains (figure 2*b*), which affords the potential to form π-stacking interactions with carbohydrate moieties. The most solvent-exposed aromatic residues lining the binding cleft are Trp46, Tyr77, Phe81 and Tyr103 on the N-terminal lobe, and Tyr292 and Phe402 on the C-terminal lobe (figure 2*b*). In addition, the electrostatic surface shows a very prominent negatively charged area in and around the ligand-binding cleft (figure 2*c*), which is similarly characteristic for carbohydrate-binding proteins. In UspC, this negative surface patch reflects a cluster of five acidic residues (Asp216, Asp270, Asp273, Glu410 and Asp414), while the acidic patch in the centre of the pocket is linked to Asp145. At the left rim of the pocket, Asp47 and Glu48 form a third prominent acidic patch (figure 2*c*). Prominent negative surface patches, although less extensive, are also seen in the substrate binding cleft of UgpB and of GacH, a UspC homologue identified by structural similarity (electronic supplementary material, figure S7). Superposition of the structures of UspC with maltotetraose-bound GacH (PDB entry 4K00 [18]) suggests that residues Asp145, Tyr292 and Gln218, which cluster in the centre of the substrate binding cleft (figure 2*b*), may play a critical role in ligand binding. These residues were subsequently subjected to a mutational analysis (see §2.5).

### 2.5. Identification of carbohydrate ligands for UspC_Nt_

**Figure 3.**
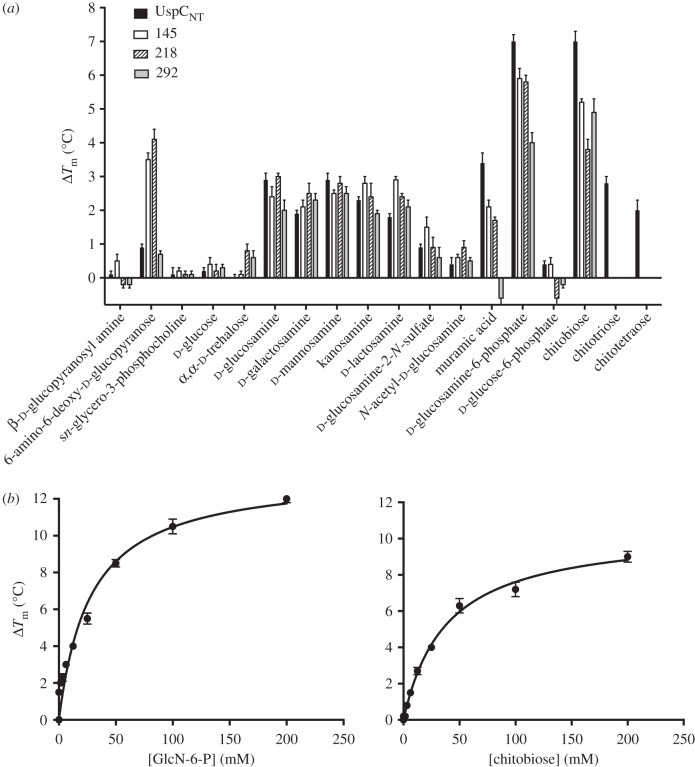
Thermal shift assay probing a panel of potential UspC ligands. (*a*) Bar graph illustrating shifts of *T*_m_ for a series of carbohydrates. Data shown are from three independent repeats. UspC_Nt_ mutants carrying a single alanine substitution at Asp145, Gln218 or Tyr292 are labelled as 145, 218 or 292, respectively. (*b*) Saturation binding curve derived from the thermal shift data varying the ligand concentration (0–200 mM). The apparent dissociation equilibrium constants *K_d,app_* derived from these data by fitting a single-site saturation binding model are 27.7 ± 6.4 mM (d-glucosamine-6-phosphate) and 38.1 ± 3.5 mM (chitobiose).

**Figure 4.**
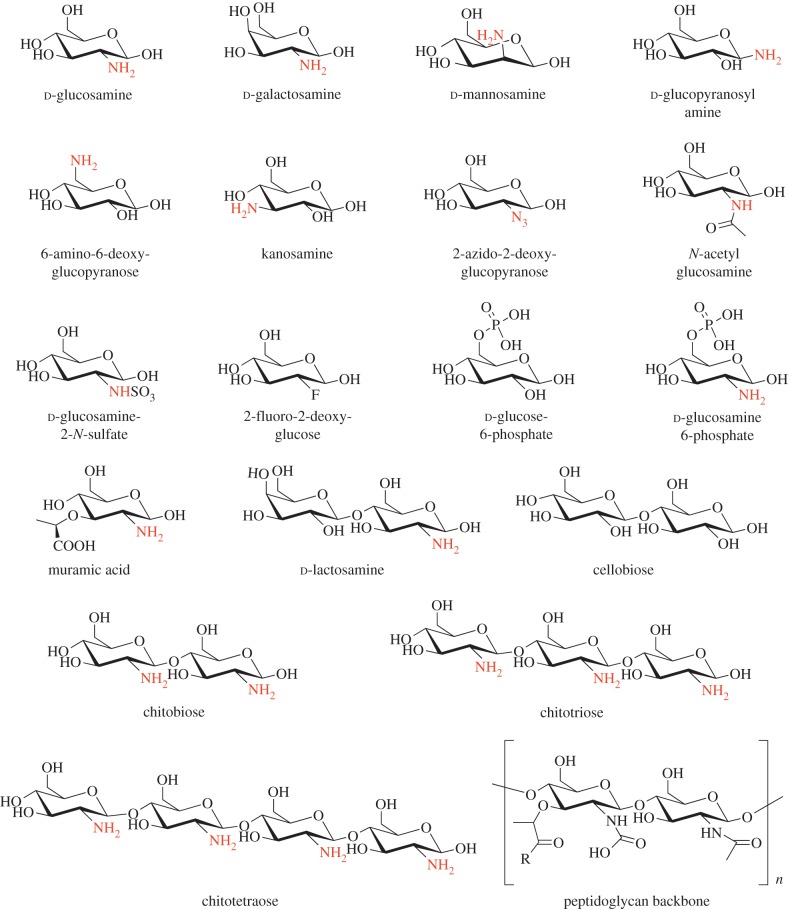
Panel of selected carbohydrates probed in the thermal shift assay. Carbohydrate ligands used in the thermal shift assay, and comparison with the PG backbone.

In order to identify ligands that bind to *Mtb-*UspC_Nt_, we tested a series of carbohydrates for their ability to stabilize the structure of UspC_Nt_ in a thermal shift assay, monitoring the shift of the melting temperature *T*_m_ in response to the addition of a diverse set of carbohydrate ligands. In total, 31 different carbohydrates were probed, ranging from mono-, to tetra-saccharides, and comprising pentose and hexose carbohydrates, amino-carbohydrates, phosphorylated carbohydrates as well as C2-modified carbohydrates (figures 3*a* and 4; electronic supplementary material, figure S7). The carbohydrates were selected on the basis that they were readily available and would provide a rational basis for a fragment-led approach to identifying important structure–function relationships of key structural components that affect binding to *Mtb-*UspC_Nt_. In figure 3*a*, we show the *T*_m_ shift of UspC_Nt_, in the presence of the respective carbohydrate (100 mM) relative to the protein alone. Strikingly, several amino-monosaccharides resulted in an increase of *T*_m_ of up to 3°C relative to the *apo* protein, including d-glucosamine, d-galactosamine and d-mannosamine (figure 3*a*). This led us to probe the importance of the amino moiety in recognition and binding to UspC_Nt_. The amino group at C2 can be tolerated in either the equatorial or axial stereoisomer (comparison of d-glucosamine with d-mannosamine, respectively (figures 3*a* and 4)). Similarly, the stereo-specificity of the hydroxyl group at C4 can be tolerated in either axial or equatorial configuration (comparison of d-glucosamine with d-galactosamine, respectively). The presence of an amino group at C1, in the case of β-d-glucopyranosyl amine, or C6, in the case of 6-amino-6-deoxy-d-glucopyranose, did not result in a significant change in the *T*_m_ of UspC_Nt_, whereas an amino group at C3, in the case of kanosamine, resulted in a shift in the *T*_m_ of UspC_Nt_ of 3°C, comparable to the C2 amino sugars d-glucosamine, d-galactosamine and d-mannosamine. Together these results indicate that an amino group at C2 or C3 is able to stabilize the structure of UspC_Nt_, suggesting that these sugars are themselves ligands or form a fragment of a ligand recognized by UspC.

Modifying the amino moiety at C2 revealed that a free amino group is an essential requirement for binding to UspC_Nt_, as the *T*_m_ of UspC_Nt_ remained unchanged or increased only moderately relative to the *apo* protein for 2-azido-2-deoxy-d-glucose, *N*-acetyl-d-glucosamine, 2-deoxy-2-fluoro-d-glucose and d-glucosamine-2-*N*-sulfate (figure 3*a* and electronic supplementary material, figure S7). Additional moieties decorating the glucosamine unit can also be tolerated, as exemplified by muramic acid (MurNAc), a lactic acid derivative of d-glucosamine, and d-glucosamine-6-phosphate, which both show positive *T*_m_ melting points shifts of 3.5°C and 7.9°C, respectively. The increased stability of d-glucosamine-6-phosphate over d-glucosamine suggests that the 6-phosphoryl group has a positive additive effect upon binding, which nonetheless remains dependent on the amino group at C2, as no increase of *T*_m_ relative to *apo* UspC_Nt_ is observed for glucose-6-phosphate.

Given that the cell wall of *Mtb* comprises peptidoglycan (PG), consisting of β(1,4)-linked disaccharide subunits of *N*-acetylated MurNAc and *N*-acetylated glucosamine (GlcNAc), we were interested to examine the commercial sugar chitobiose, a disaccharide of β-1,4-linked d-glucosamine units that, apart from *N*-acetylation, mimics the carbohydrate backbone of PG (figure 4). Addition of chitobiose to UspC_Nt_ did indeed result in an increase of *T*_m_ by 6.7°C, a shift greater than that afforded by the monosaccharides MurNAc or glucosamine alone. By contrast, the addition of d-lactosamine, a disaccharide comprising d-galactose in β(1,4)-linkage with d-glucosamine, resulted in a shift that is comparable to that of the mono-saccharide d-glucosamine. These results indicate that both of the C2 amino groups of chitobiose have a positive role in binding and substrate recognition to UspC_Nt_. One hallmark feature identified from these binding studies is that increasing the length of the d-glucosamine oligosaccharide to tri- and tetra-β-1,4-linked d-glucosamine units in the case of chitotriose and chitotetraose significantly reduced the binding of these carbohydrates to UspC_Nt_, in comparison to chitobiose, suggesting that binding and recognition is dependent upon the length of the carbohydrate.

Overall, our thermal shift assay identified chitobiose and d-glucosamine-6-phosphate as ligands with the greatest effect on the stability of UspC_Nt_. Therefore, these ligands were used to examine the dose-dependence of stabilization. We found that *ΔT*_m_ showed saturation binding behaviour in response to the addition of these amino sugars, allowing us to determine an apparent binding affinity *K_d,app_* of 27 mM and 38 mM for d-glucosamine-6-phosphate and chitobiose, respectively (figure 3*b*).

We were not successful in co-crystallizing UspC_Nt_ with chitobiose, d-glucosamine-6-phosphate or d-glucosamine. However, the superposition of UspC_Nt_ with carbohydrate-bound GacH had suggested a potential role for Asp145, Gln218 and Tyr292 in ligand binding. We therefore generated point mutants of UspC_Nt_ where these side chains were substituted by alanine. Monitoring the shift in *T*_m_ of these point mutants against our panel of carbohydrates shows a similar profile of changes in *T*_m_ as wild-type UspC_Nt_ (figure 3*a*). However, a reduction in shift in *T*_m_ of UspC_Nt_ of the d-glucosamine-6-phosphate and chitobiose was observed in the cases of the Asp145Ala, Gln218Ala and Tyr292Ala, supporting the notion that these residues do play a role in substrate selectivity.

### 2.6. Saturation transfer difference-NMR of UspC_Nt_ with d-glucosamine and chitobiose

**Figure 5.**
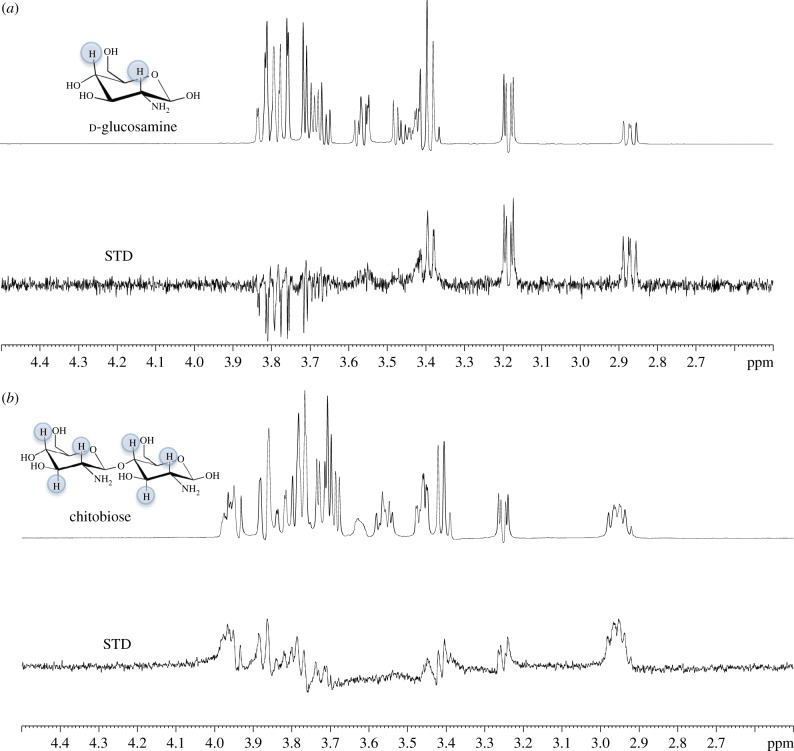
STD-NMR analysis of amino-sugar binding to UspC_Nt_. d-Glucosamine (50 mM) (*a*) and chitobiose (15 mM) (*b*) were probed using ^1^H-NMR in the absence (top) and presence (bottom) of 30 µM UspC_Nt_. The top panel in both (*a*) and (*b*) represent the reference NMR spectrum, while the bottom panel in both (*a*) and (*b*) represents the STD-NMR spectrum. The STD effects of the protons involved for each amino sugar are highlighted (blue).

To gain insight into the molecular basis of carbohydrate recognition saturation transfer difference (STD)-NMR was employed with UspC_Nt_ and the identified d-glucosamine and chitobiose ligands from the thermal shift assays to characterize the epitope of the carbohydrate that is involved in binding to UspC_Nt_. STD effects were observed in the STD-NMR difference spectrum for both d-glucosamine (50 mM) and chitobiose (15 mM) and hence provide further support that both of these amino sugars do bind to the UspC_Nt_ protein (figure 5). From these experiments, we have determined from the STD-NMR binding pattern that the H2 (α and β) and H4 (α and β) of d-glucosamine interact non-covalently with *Mtb*-UspC_Nt_ (figure 5*a*) and that in the case of chitobiose the H2 (α and β), H3 (α and β) and H4 (α and β) interact with *Mtb*-UspC_Nt_ (figure 5*b*). It has not been possible to determine which subunit of the β-1,4-linked d-glucosamine units of chitobiose is involved without higher resolution NMR. Given the additional interaction observed with chitobiose in the STD-NMR experiment, this goes some way to explaining the increased *T*_m_ shift observed from the thermal shift assays.

## 3. Discussion

To date, the nutrient requirements of *Mtb* during infection inside the human host remain to be fully elucidated [11]. Remarkably, there is little known regarding the identity and properties and mechanisms of the proteins that are involved in the import of essential nutrients. The structural and biochemical analysis of carbohydrate transport systems are an important step towards understanding how *Mtb* can acquire essential nutrients from a carbohydrate-limited host cell environment.

Our X-ray crystallographic structure determination revealed that UspC has the same overall fold and architecture as other carbohydrate-binding proteins associated with characterized ABC transporter systems [15,18], comprising two subdomains joined by a central hinge region that enclose the (putative) substrate binding cleft. Structural similarity to the solute-binding unit of the *E. coli* maltose transporter [26], the capacity for conformational flexibility between N- and C-terminal lobes (figure 1*b*), distribution of solvent-accessible aromatic side chains in the binding cleft and the characteristic acidic molecular surface (figure 2*b*) are structural features fully consistent with the proposed role as the substrate binding unit of the carbohydrate UspABC ABC transporter system.

The thermal shift data have provided the first evidence for carbohydrate-binding selectivity of UspC (figure 3; electronic supplementary material, figure S8). Among the panel of carbohydrates tested, there is a clear preference for sugars with a free amino group at C2, whereby adding a phosphate at C6 to d-glucosamine or using the amino-disaccharide chitobiose markedly increased the thermal stability of UspC_Nt_. Although the *in vitro* binding affinities of these two ligands are relatively weak to *Mtb*-UspC_Nt_, when compared with the affinity of *sn*-glycero-3-phosophocholine for UgpB (*K_d_* ∼ 27 µM) of the UgpABCE transporter system [15], binding affinities of up to 8 mM have been reported for a PG recognition lectin with mimics of the PG backbone [28]. Nonetheless, the STD-NMR data have revealed the specific binding epitope for d-glucosamine and chitobiose (figure 5); however, this does not preclude the potential for the further identification of higher affinity substrates.

Recycling and import of saccharides and lipids are emerging as an essential feature of survival of *Mtb* in macrophages. A prime example is the LpqY-SugABC system, which has been implicated in recycling of trehalose [13], the saccharide component of the primary mycobacterial cell-wall lipid cord factor [29]. Similarly, the UgpABCE transporter system has been implicated in importing the lipid *sn*-glycero-3-phosphocholine [15]. Consistent with low sequence identity between UspC and LpqY (17%) or UgpB (22%), neither trehalose nor *sn*-glycero-3-phosphocholine increase the stability of UspC_Nt_ (figure 3), reinforcing the notion that carbohydrate transport permease systems in mycobacteria have defined substrate preferences. Such preferences are also manifested in that relatively subtle changes of structural features in the ligand-binding cleft can have a pronounced effect on ligand affinity. For instance, binding of *Mtb* UgpB to *sn*-glycero-3-phosphocholine depends on the presence of solvent-exposed Leu205 in the active site cleft, in line with the hydrophobic nature of the intact substrate [15]. Even when Leu205 is substituted with the corresponding tryptophan from the *E. coli* UgpB orthologue, glycerol-3-phosphate binding is not restored [15]. When mapped onto the structure of UspC_Nt_, Leu205 of *Mtb* UgpB falls close to Gln218, one of the active site cleft residues mutation of which markedly affected the stabilizing effect of chitobiose.

Given the potential lack of availability of diverse carbohydrates during intracellular infection of *Mtb* within the environment of the phagosome, our findings that UspC preferentially binds amino sugars are significant. While, to our knowledge, chitobiose is not found within the phagosome, the structural relationship of the binder chitobiose to the PG backbone of the mycobacterial cell wall is striking (figure 4). The UspABC transporter is likely to be localized in the inner-membrane of the cell wall, with the solute-binding UspC protein positioned in the periplasmic space between the inner-membrane and the mycolic acid–arabinogalactan–peptidoglycan core of the mycobacterial cell wall, thus positioning UspC in close proximity to cell-wall amino-sugar substrates. Amino sugars are abundant in the cell wall of *Mtb*, not least as the dominant component of cell-wall PG, for which the UspC-binder chitobiose can be considered a deacetylated analogue. Similarly, d-galactosamine is present through the modification of interior branched arabinosyl residues in the arabinogalactan layer [30]. It could therefore be envisaged that from a physiological stand point, UspC requires relatively high binding affinities for its amino-sugar substrates to prevent the organism from depriving the integral cell wall of amino sugars unless required. If PG were the origin of UspABC substrates, transport would probably require de-acetylation, which could be mediated by *Mtb* Rv1096, known to deacetylate PG [31], or through additional yet to be identified deacetylases [30]. Hydrolysis of PG is known to be mediated through the lytic transglycosylase resuscitation promoting factors (Rpf) that cleave the glycosidic β-(1,4)-linkage between alternating MurNAc–GlcNAc residues resulting in disaccharide functional units [32]. It is therefore tantalizing to link the essentiality of the UspABC transporter [19] to a potential functional role in recycling of amino-sugar cell-wall components, thus contributing to evolutionary adaptation to the carbohydrate-limited niche of host macrophages and optimizing the use of scarce carbohydrates within this environmental niche. Further experiments are now underway to further investigate this hypothesis.

In conclusion, our data strongly indicate that *Mtb*-UspC is a carbohydrate-binding unit of the essential UspABC transporter system, with a substrate preference for sugars containing an amino group at the C2 or C3 position. These data indicate a potential functional role for the *Mtb* UspABC transport system in recycling key components of PG from the mycobacterial cell wall, affording *Mtb* the opportunity to use scarce nutrients during intracellular infection.

## 4. Material and methods

All chemicals and reagents were purchased from Sigma-Aldrich, with the exception of all of the carbohydrates used in this study, which were purchased from Carbosynth. Restriction enzymes were obtained from New England Biolabs. Double-distilled water was used throughout.

### 4.1. Plasmid constructs, protein expression and purification

Full length and an N-terminal truncated form (codons 31– 440) of *Mtb uspC* were amplified from *Mtb* genomic DNA and cloned into either pET28a (+) or pET23b (+) vector using the NdeI and HindIII restriction enzyme sites resulting in the constructs: *uspC_pet28a, uspC_pet23b, uspC_T_pet28a* and *uspC_T_pet23b*. The primer sequences are listed in the electronic supplementary material, table S2. Targeted single-site substitutions were introduced into *uspC_T_pet28a* using the primers listed in the electronic supplementary material, table S2 with Phusion Polymerase and the PCR cycle (98°C, 30 s; 20 cycles of 98°C, 30 s; 60°C, 30 s; 72°C, 4 min; followed by 5 min at 72°C), followed by digestion with 1 µl DpnI. Plasmid sequences were verified and used for protein expression.

*Escherichia coli* BL21(DE3) competent cells were transformed with the *uspC* expression plasmid, grown at 27°C to an optical density at 600 nm (OD_600_) of 0.8–1.0 in Terrific Broth medium (Difco) supplemented with either 50 µg ml^–1^ kanamycin (pET28a constructs) or 100 µg ml^−1^ ampicillin (pET23b constructs). Protein production was induced with 1 mM isopropyl-β-thiogalactopyranoside (IPTG) and the cultures were grown at 16°C overnight with shaking. The cells were harvested and resuspended in lysis buffer (50 mM NaH_2_PO_4_, 300 mM NaCl, 10% glycerol pH 7.6 (buffer A) supplemented with 0.1% Triton-X 100 and Complete Protease Inhibitor Cocktail (Roche). The cells were freeze–thawed and sonicated on ice (Sonicator Ultrasonic Liquid Processor XL; Misonix). Following centrifugation (27 000*g*, 40 min, 4°C) the supernatant was loaded onto Ni^2+^-affinity resin (Qiagen). Recombinant UspC was eluted from the Ni^2+^-affinity column in buffer A with increasing concentrations of imidazole. Fractions containing the protein were dialysed against 20 mM Tris–HCl, 100 mM NaCl, 10% glycerol pH 8.0 (buffer B). After dialysis, the protein was loaded onto a 1 ml QHP ion exchange column (GE Healthcare) and eluted with buffer B with increasing NaCl concentrations (0.1–1 M). Fractions containing pure UspC were dialysed at 4°C against 50 mM HEPES, 100 mM NaCl, 5% glycerol, pH 7.6. The identity of the protein was confirmed by tryptic digest and nanoLC-ESI-MS/MS (WPH Proteomics Facility, University of Warwick).

### 4.2. Crystallization and structure determination

Purified UspC (truncated at the N-terminus to remove the first 31 amino acids: UspC_Nt_) was concentrated by ultrafiltration (30 kDa cutoff; Amicon Ultra) to 10 mg ml^−1^ in 50 mM HEPES, 100 mM NaCl, 5% glycerol, pH 7.6. Crystals were grown by vapour diffusion in 96-well, sitting-drop plates (SwissSci), using an automatic liquid handling system (Mosquito, TTP Labtech) to pipette drops of 150 nl protein solution mixed with 150 nl reservoir solution. Reservoir conditions producing diffracting crystals are listed in the electronic supplementary material, table S3. Crystals appeared after 1–3 days at 18°C. UspC crystals were mounted into nylon loops directly from the crystallization drop and flash-frozen in liquid nitrogen prior to data collection.

Diffraction data were recorded for two crystal forms (table 1) at the Diamond Light Source, respectively. Initial phases were determined based on an iodine derivative, using single-wavelength anomalous diffraction data recorded on our in-house source (Rigaku MicroMax007HF, VariMax optics, Saturn 944 CCD). All diffraction data were integrated and scaled using XDS, XSCALE [33] and programs of the CCP4 suite [34]. Heavy atom positions were determined in SHELXD [35], and phases calculated in SHARP [36], followed by solvent-flattening in SOLOMON [37]. An initial model of UspC was generated using ARP/wARP [38], extended manually (COOT [39]) and used to determine a molecular replacement solution for the monoclinic crystal form (PHASER [40]). An improved experimental density map could be generated by multi-crystal averaging (DMMULTI [34]), which allowed to build and refine (REFMAC5 [41], PHENIX.REFINE [42]) a complete structural model. The final refined model comprises residues 34–441 of the sequence of *Mtb*-UspC, plus an additional six residues originating from the C-terminal affinity tag encoded by the expression plasmid. Crystallographic data and refinement statistics are shown in table 1. Figures were prepared using PyMol (www.pymol.org) adopting the Corey–Pauling–Koltun (CPK) colouring scheme: O, red; N, blue; S, yellow and C, green, as indicated in the figure legends.

### 4.3. Deposition of coordinates and structure factors

Coordinates and structure factors have been deposited in the Protein Data Bank under PDB accession codes 5K2X (tetragonal crystal form) and 5K2Y (monoclinic crystal form).

### 4.4. Protein thermal shift assay

The transition unfolding temperature *T*_m_ of the UspC_Nt_ protein (30 µM) was determined in the presence or the absence of ligands. The carbohydrate screen used a constant ligand concentration of 100 mM, while the saturation binding experiment probed *T*_m_ over a concentration range from 0 to 200 mM. Reactions were performed in a total volume of 20 µl using Rotor-Gene Q Detection System (Qiagen), setting the excitation wavelength to 470 nm and detecting emission at 557 nm of the SYPRO Orange protein gel stain, 15 × final concentration (Invitrogen). The cycle used was a melt ramp from 30 to 95°C, increasing temperature in 1°C steps and time intervals of 5 s. Fluorescence intensity was plotted as a function of temperature. The *T*_m_ was determined using the Rotor-Gene Q software and the Analysis Melt functionality. All experiments were performed in triplicate. To obtain saturation binding data, the Δ*T*_m_ values of two experiments were averaged and plotted against concentration of compound. *K_d,app_* was determined by fitting a single-site binding model (GraphPad, Prism 5).

### 4.5. Saturation transfer difference NMR

UspC was buffer exchanged into deuterated phosphate-buffered saline (PBS) and the ligands dissolved in deuterated PBS. All STD-NMR experiments were recorded on a 600 MHz Bruker Avance III instrument equipped with a 5 mm TBI probe. Acquisitions were performed at 298 K using the standard STD pulse sequence with a shaped Q5 pulse train (50 ms, 90°, 4 µs delay between pulses) for selective protein irradiation, and an alteration between on and off resonances. Presaturation of the protein resonances was performed with an on-resonance radiation at 0.98 ppm; off resonance radiation was applied at 50.0 ppm where no NMR resonances of protein or ligand are present. STD spectra were recorded as described previously [43], with water suppression. A total number of 16 scans were collected for each experiment.

## Supplementary Material

Supplementary Information - combined

## Data accessibility

The datasets supporting this article have been uploaded as part of the electronic supplementary material.

## References

[1] WHO. 2015 *Global Tuberculosis Report* 2015. Geneva, Switzerland: WHO.

[2] Dheda K, Gumbo T, Gandhi NR, Murray M, Theron G, Udwadia Z, Migliori GB, Warren R 2014 Global control of tuberculosis: from extensively drug-resistant to untreatable tuberculosis. Lancet Respir. Med. 2, 321–338. (doi:10.1016/S2213-2600(14)70031-1) 24717628 10.1016/S2213-2600(14)70031-1 PMC5526327

[3] Kim DH et al. 2008 Treatment outcomes and long-term survival in patients with extensively drug-resistant tuberculosis. Am. J. Respir. Crit. Care Med. 178, 1075–1082. (doi:10.1164/rccm.200801-132OC) 18703792 10.1164/rccm.200801-132OC

[4] de Chastellier C 2009 The many niches and strategies used by pathogenic mycobacteria for survival within host macrophages. Immunobiology 214, 526–542. (doi:10.1016/j.imbio.2008.12.005) 19261352 10.1016/j.imbio.2008.12.005

[5] Cole ST et al. 1998 Deciphering the biology of *Mycobacterium tuberculosis* from the complete genome sequence. Nature 393, 537–544. (doi:10.1038/31159) 9634230 10.1038/31159

[6] Schnappinger D et al. 2003 Transcriptional adaptation of *Mycobacterium tuberculosis* within macrophages: insights into the phagosomal environment. J. Exp. Med. 198, 693–704. (doi:10.1084/jem.20030846) 12953091 10.1084/jem.20030846 PMC2194186

[7] Pandey AK, Sassetti CM 2008 Mycobacterial persistence requires the utilization of host cholesterol. Proc. Natl Acad. Sci. USA 105, 4376–4380. (doi:10.1073/pnas.0711159105) 18334639 10.1073/pnas.0711159105 PMC2393810

[8] Yam KC et al. 2009 Studies of a ring-cleaving dioxygenase illuminate the role of cholesterol metabolism in the pathogenesis of *Mycobacterium tuberculosis*. PLoS Pathog. 5, e1000344 (doi:10.1371/journal.ppat.1000344) 19300498 10.1371/journal.ppat.1000344 PMC2652662

[9] Braibant M, Gilot P, Content J 2000 The ATP binding cassette (ABC) transport systems of *Mycobacterium tuberculosis*. FEMS Microbiol. Rev. 24, 449–467. (doi:10.1111/j.1574-6976.2000.tb00550.x) 10978546 10.1111/j.1574-6976.2000.tb00550.x

[10] Titgemeyer F et al. 2007 A genomic view of sugar transport in *Mycobacterium smegmatis* and *Mycobacterium tuberculosis*. J. Bacteriol. 189, 5903–5915. (doi:10.1128/JB.00257-07) 17557815 10.1128/JB.00257-07 PMC1952047

[11] Niederweis M 2008 Nutrient acquisition by mycobacteria. Microbiology 154, 679–692. (doi:10.1099/mic.0.2007/012872-0) 18310015 10.1099/mic.0.2007/012872-0

[12] Sassetti CM, Rubin EJ 2003 Genetic requirements for mycobacterial survival during infection. Proc. Natl Acad. Sci. USA 100, 12 989–12 994. (doi:10.1073/pnas.2134250100) 10.1073/pnas.2134250100 PMC240732 14569030

[13] Kalscheuer R, Weinrick B, Veeraraghavan U, Besra GS, Jacobs WR Jr 2010 Trehalose-recycling ABC transporter LpqY-SugA-SugB-SugC is essential for virulence of *Mycobacterium tuberculosis*. Proc. Natl Acad. Sci. USA 107, 21 761–21 766. (doi:10.1073/pnas.1014642108) 10.1073/pnas.1014642108 PMC3003129 21118978

[14] Rengarajan J, Bloom BR, Rubin EJ 2005 Genome-wide requirements for *Mycobacterium tuberculosis* adaptation and survival in macrophages. Proc. Natl Acad. Sci. USA 102, 8327–8332. (doi:10.1073/pnas.0503272102) 15928073 10.1073/pnas.0503272102 PMC1142121

[15] Jiang D, Zhang Q, Zheng Q, Zhou H, Jin J, Zhou W, Bartlam M, Rao Z 2014 Structural analysis of *Mycobacterium tuberculosis* ATP-binding cassette transporter subunit UgpB reveals specificity for glycerophosphocholine. FEBS J. 281, 331–341. (doi:10.1111/febs.12600) 24299297 10.1111/febs.12600

[16] Somashekar BS, Amin AG, Rithner CD, Troudt J, Basaraba R, Izzo A, Crick DC, Chatterjee D 2011 Metabolic profiling of lung granuloma in *Mycobacterium tuberculosis* infected guinea pigs: ex vivo ^1^H magic angle spinning NMR studies. J. Proteome Res. 10, 4186–4195. (doi:10.1021/pr2003352) 21732701 10.1021/pr2003352

[17] Lew JM, Kapopoulou A, Jones LM, Cole ST 2011 TubercuList--10 years after. Tuberculosis 91, 1–7. (doi:10.1016/j.tube.2010.09.008) 20980199 10.1016/j.tube.2010.09.008

[18] Vahedi-Faridi A, Licht A, Bulut H, Scheffel F, Keller S, Wehmeier UF, Saenger W, Schneider E 2010 Crystal structures of the solute receptor GacH of *Streptomyces glaucescens* in complex with acarbose and an acarbose homolog: comparison with the acarbose-loaded maltose-binding protein of *Salmonella typhimurium*. J. Mol. Biol. 397, 709–723. (doi:10.1016/j.jmb.2010.01.054) 20132828 10.1016/j.jmb.2010.01.054

[19] Griffin JE, Gawronski JD, Dejesus MA, Ioerger TR, Akerley BJ, Sassetti CM 2011 High-resolution phenotypic profiling defines genes essential for mycobacterial growth and cholesterol catabolism. PLoS Pathog. 7, e1002251 (doi:10.1371/journal.ppat.1002251) 21980284 10.1371/journal.ppat.1002251 PMC3182942

[20] Cole ST et al. 2001 Massive gene decay in the leprosy bacillus. Nature 409, 1007–1011. (doi:10.1038/35059006) 11234002 10.1038/35059006

[21] Krogh A, Larsson B, von Heijne G, Sonnhammer EL 2001 Predicting transmembrane protein topology with a hidden Markov model: application to complete genomes. J. Mol. Biol. 305, 567–580. (doi:10.1006/jmbi.2000.4315) 11152613 10.1006/jmbi.2000.4315

[22] Bendtsen JD, Nielsen H, von Heijne G, Brunak S 2004 Improved prediction of signal peptides: SignalP 3.0. J. Mol. Biol. 340, 783–795. (doi:10.1016/j.jmb.2004.05.028) 15223320 10.1016/j.jmb.2004.05.028

[23] Davis IW, Murray LW, Richardson JS, Richardson DC 2004 MOLPROBITY: structure validation and all-atom contact analysis for nucleic acids and their complexes. Nucleic Acids Res. 32, W615–W619. (doi:10.1093/nar/gkh398) 15215462 10.1093/nar/gkh398 PMC441536

[24] Krissinel E, Henrick K 2004 Secondary-structure matching (SSM), a new tool for fast protein structure alignment in three dimensions. Acta Crystallogr. D Biol. Crystallogr. 60, 2256–2268. (doi:10.1107/S0907444904026460) 15572779 10.1107/S0907444904026460

[25] Krissinel E, Henrick K 2007 Inference of macromolecular assemblies from crystalline state. J. Mol. Biol. 372, 774–797. (doi:10.1016/j.jmb.2007.05.022) 17681537 10.1016/j.jmb.2007.05.022

[26] Oldham ML, Chen J 2011 Snapshots of the maltose transporter during ATP hydrolysis. Proc. Natl Acad. Sci. USA 108, 15 152–15 156. (doi:10.1073/pnas.1108858108) 10.1073/pnas.1108858108 PMC3174604 21825153

[27] McNicholas S, Potterton E, Wilson KS, Noble ME 2011 Presenting your structures: the CCP4 *mg* molecular-graphics software. Acta Crystallogr. D Biol. Crystallogr. 67, 386–394. (doi:10.1107/S0907444911007281) 21460457 10.1107/S0907444911007281 PMC3069754

[28] Lehotzky RE, Partch CL, Mukherjee S, Cash HL, Goldman WE, Gardner KH, Hooper LV 2010 Molecular basis for peptidoglycan recognition by a bactericidal lectin. Proc. Natl Acad. Sci. USA 107, 7722–7727. (doi:10.1073/pnas.0909449107) 20382864 10.1073/pnas.0909449107 PMC2867859

[29] Noll H, Bloch H, Asselineau J, Lederer E 1956 The chemical structure of the cord factor of *Mycobacterium tuberculosis*. Biochim. Biophys. Acta 20, 299–309. (doi:10.1016/0006-3002(56)90289-X) 13328853 10.1016/0006-3002(56)90289-x

[30] Skovierova H et al. 2010 Biosynthetic origin of the galactosamine substituent of arabinogalactan in *Mycobacterium tuberculosis*. J. Biol. Chem. 285, 41 348–41 355. (doi:10.1074/jbc.M110.188110) 10.1074/jbc.M110.188110 PMC3009860 21030587

[31] Yang S, Zhang F, Kang J, Zhang W, Deng G, Xin Y, Ma Y 2014 *Mycobacterium tuberculosis* Rv1096 protein: gene cloning, protein expression, and peptidoglycan deacetylase activity. BMC Microbiol. 14, 174 (doi:10.1186/1471-2180-14-174) 24975018 10.1186/1471-2180-14-174 PMC4087242

[32] Kana BD et al. 2008 The resuscitation-promoting factors of *Mycobacterium tuberculosis* are required for virulence and resuscitation from dormancy but are collectively dispensable for growth *in vitro*. Mol. Microbiol. 67, 672–684. (doi:10.1111/j.1365-2958.2007.06078.x) 18186793 10.1111/j.1365-2958.2007.06078.x PMC2229633

[33] Kabsch W 2010 Integration, scaling, space-group assignment and post-refinement. Acta Crystallogr. D Biol. Crystallogr. 66, 133–144. (doi:10.1107/S0907444909047374) 20124693 10.1107/S0907444909047374 PMC2815666

[34] Winn MD et al. 2011 Overview of the *CCP4* suite and current developments. Acta Crystallogr. D Biol. Crystallogr. 67, 235–242. (doi:10.1107/S0907444910045749) 21460441 10.1107/S0907444910045749 PMC3069738

[35] Sheldrick GM 2008 A short history of SHELX. Acta Crystallogr. A Found Adv. 64, 112–122. (doi:10.1107/S0108767307043930) 10.1107/S0108767307043930 18156677

[36] Bricogne G, Vonrhein C, Flensburg C, Schiltz M, Paciorek W 2003 Generation, representation and flow of phase information in structure determination: recent developments in and around SHARP 2.0. Acta Crystallogr. D Biol. Crystallogr. 59, 2023–2030. (doi:10.1107/S0907444903017694) 14573958 10.1107/s0907444903017694

[37] Abrahams JP, Leslie AG 1996 Methods used in the structure determination of bovine mitochondrial F1 ATPase. Acta Crystallogr. D Biol. Crystallogr. 52, 30–42. (doi:10.1107/S0907444995008754) 15299723 10.1107/S0907444995008754

[38] Langer G, Cohen SX, Lamzin VS, Perrakis A 2008 Automated macromolecular model building for X-ray crystallography using ARP/wARP version 7. Nat. Protoc. 3, 1171–1179. (doi:10.1038/nprot.2008.91) 18600222 10.1038/nprot.2008.91 PMC2582149

[39] Emsley P, Lohkamp B, Scott WG, Cowtan K 2010 Features and development of Coot. Acta Crystallogr. D Biol. Crystallogr. 66, 486–501. (doi:10.1107/S0907444910007493) 20383002 10.1107/S0907444910007493 PMC2852313

[40] McCoy AJ, Grosse-Kunstleve RW, Adams PD, Winn MD, Storoni LC, Read RJ 2007 Phaser crystallographic software. J. Appl. Crystallogr. 40, 658–674. (doi:10.1107/S0021889807021206) 19461840 10.1107/S0021889807021206 PMC2483472

[41] Murshudov GN, Skubák P, Lebedev AA, Pannu NS, Steiner RA, Nicholls RA, Winn MD, Long F, Vagin AA 2011 *REFMAC5* for the refinement of macromolecular crystal structures. Acta Crystallogr. D Biol. Crystallogr. 67, 355–367. (doi:10.1107/S0907444911001314) 21460454 10.1107/S0907444911001314 PMC3069751

[42] Adams PD et al. 2010 PHENIX: a comprehensive Python-based system for macromolecular structure solution. Acta Crystallogr. D Biol. Crystallogr. 66, 213–221. (doi:10.1107/S0907444909052925) 20124702 10.1107/S0907444909052925 PMC2815670

[43] Castro S, Duff M, Snyder NL, Morton M, Kumar CV, Peczuh MW 2005 Recognition of septanose carbohydrates by concanavalin A. Org. Biomol. Chem. 3, 3869–3872. (doi:10.1039/b509243d) 16239999 10.1039/b509243d

