# Supplementary material for: Structural and functional analysis of the solute-binding protein UspC from Mycobacterium tuberculosis that is specific for amino sugars
Source: Open Biol. 2016 Jun 22;6(6):160105. doi: 10.1098/rsob.160105 (PMC4929945; doi:10.1098/rsob.160105)

Structural and Functional Analysis of the Solute-binding Protein UspC from *Mycobacterium tuberculosis* that is specific for amino sugars

Electronic supplementary material

Elizabeth Fullam[a,b], Ivan Prokes[c] Klaus Fütterer[a] and Gurdyal S. Besra[a]

[a] School of Biosciences, University of Birmingham, Edgbaston, Birmingham, B15 2TT, UK

[b] Current address School of Life Sciences, University of Warwick, Coventry, CV4 7AL UK

[c] Department of Chemistry, University of Warwick, Coventry, CV4 7AL, UK

Authors for correspondence:

Elizabeth Fullam:
e-mail: e.fullam@warwick.ac.uk

Gurdyal Besra:
e-mail: g.besra@bham.ac.uk

The datasets supporting this article have been uploaded as part of the supplementary material.

Supplementary tables S1 – S3

Table S1. Amino acid sequence identity (%) between mycobacterial UspC orthologues.

	Mtb_Uspc	Mbovis_UspC	Mcanetti_UspC	Mmarinum_UpsC	Mulcerans_UspC	Mkanassi_UspC	Mleprae_UpsC	Mintracellulare_U	Msmeg_UspC
Mtb_Uspc		99.77	99.32	81.82	81.59	83.18	75.68	78.64	70.12
Mbovis_UspC	99.77		99.09	81.59	81.36	82.95	75.45	78.41	70.35
Mcanetti_UspC	99.32	99.09		81.36	81.59	83.18	75.45	78.64	69.88
Mmarinum_UpsC	81.82	81.59	81.36		99.32	84.62	72.17	76.02	68.15
Mulcerans_UspC	81.59	81.36	81.59	99.32		84.84	71.95	76.24	68.15
Mkanassi_UspC	83.18	82.95	83.18	84.62	84.84		74.21	76.7	69.32
Mleprae_UpsC	75.68	75.45	75.45	72.17	71.59	74.21		73.26	66.05
Mintracellulare_UspC	78.64	78.41	78.64	76.02	76.24	76.7	73.26		72.09
Msmeg_UspC	70.12	70.35	69.88	68.15	68.15	69.32	66.05	72.09	

ClustalW2 (http://www.ebi.ac.uk/clustalw/) was used to score alignments between full length UspC from *Mtb* with orthologues from *M. bovis, M. canetti, M. marinum, M. ulcerans, M. kanassi, M. leprae, M. intracellulare* and *M. smegmatis*.

Table S2: Oligonucleotides used for recombinant expression plasmids. Restriction recognition sites are underlined. Codon encoding the amino acid mutation is indicated in bold type

Name	Use	Sequence (5'-3')
UspC_FL_F_NHis	Clone full length UspC into pET28a	AAAAAACATATGGCCGAACCGCGTGGCGGAAAG
UspC_FL_R_NHis	Clone full length UspC into pET28a	AAAAAAGCTTCTAGCGCTGTGTGGCAGCATTG
UspC_FL_F_CHis	Clone full length UspC into pET23b	AAAAAACATATGGCCGAACCGCGTGGCGGAAAG
UspC-FL_R-CHis	Clone full length UspC into pET23b	AAAAAAGCTTGCGCTGTGTGGCAGCATTG
UspC_T_F_NHis	Clone truncated UspC into pET28a	AAAAAACATATGGCCGAACCGCGTGGCGGAAAG
UspC_T_R_NHis	Clone truncated UspC into pET28a	AAAAAAGCTTCTAGCGCTGTGTGGCAGCATTG
UspC_T_F_CHis	Clone truncated UspC into pET23b	AAAAAACATATGGCCGAACCGCGTGGCGGAAAG
UspC_T_R_CHis	Clone truncated UspC into pET23b	AAAAAAGCTTGCGCTGTGTGGCAGCATTG
UspC_145SDM_5	Mutate residue ASP145ALA	CCGCAACTGACG**GCC**GCCGGAATTGCC
UspC_145SDM_3	Mutate residue ASP145ALA	GGCAATTCCGGC**GGC**CGTCAGTTGCGG
UspC_218SDM_5	Mutate residue GLN218ALA	GCCGCCAACGATCCT**GCG**GCCATCTACCTTAAC
UspC_218SDM_3	Mutate residue GLN218ALA	GTTAAGGTAGATGGC**CGC**AGGATCGTTGGCGGC
UspC_292SDM_5	Mutate residue TYR292ALA	CCAGTCCGGCACC**GCC**AGTTTGGCGCCG
UspC_292SDM_3	Mutate residue TYR292ALA	CGGCGCCAAACTGG**CGG**TGCCGGACTGG

Table S3. Reservoir conditions for vapour diffusion crystallization of UspC. The commercial sparse matrix screen Morpheus (Molecular Dimensions) was used to obtain crystals.

**$UspC_{Nt}$ – apo, tetragonal**	**$UspC_{Nt}$ – apo, monoclinic**	**$UspC_{Nt}$ – iodine derivative**
Morpheus Condition D4<br><br>0.12 M Alcohols<br><br>0.1 M Buffer System 1<br><br>50 % v/v precipitant Mix 4 pH 6.5	Morpheus condition B4<br><br>0.09 M Halogens<br><br>0.1 M Buffer System 1<br><br>50 % v/v Precipitant Mix 4 pH 6.5	Morpheus Condition D8<br><br>0.12 M Alcohols<br><br>0.1 M Buffer System 2<br><br>50 % v/v Precipitant Mix 4 pH 7.5

Alcohols: 1,6-Hexanediol; 1-Butanol; 1,2-Propanediol; 2-Propanol; 1,4-Butanediol; 1,3-Propanediol

Halogens: Sodium fluoride; Sodium bromide; Sodium iodide

Buffer System 1: 1 M Imidazole; MES monohydrate (acid)

Buffer System 2: 1 M Sodium HEPES; MOPS (acid)

Precipitant Mix 4: 25% v/v MPD; 25% PEG 1000; 25% w/v PEG 3350

References for electronic supplementary material

1. Robert X, Gouet P 2014 Deciphering key features in protein structures with the new ENDscript server. *Nucleic Acids Res* **42**, W320-W324. (10.1093/nar/gku316)

2. Vahedi-Faridi A, Licht A, Bulut H, Scheffel F, Keller S, Wehmeier UF, Saenger W, Schneider E 2010 Crystal structures of the solute receptor GacH of Streptomyces glaucescens in complex with acarbose and an acarbose homolog: comparison with the acarbose-loaded maltose-binding protein of Salmonella typhimurium. *J Mol Biol* **397**, 709-723. (10.1016/j.jmb.2010.01.054)

3. Oldham ML, Chen J 2011 Snapshots of the maltose transporter during ATP hydrolysis. *Proc Natl Acad Sci U S A* **108**, 15152-15156. (10.1073/pnas.1108858108)

4. Jiang D, Zhang Q, Zheng Q, Zhou H, Jin J, Zhou W, Bartlam M, Rao Z 2014 Structural analysis of Mycobacterium tuberculosis ATP-binding cassette transporter subunit UgpB reveals specificity for glycerophosphocholine. *FEBS J* **281**, 331-341. (10.1111/febs.12600)

5. McNicholas S, Potterton E, Wilson KS, Noble ME 2011 Presenting your structures: the CCP4mg molecular-graphics software. *Acta Crystallogr D Biol Crystallogr* **67**, 386-394. (10.1107/S0907444911007281)

Figure S1. Sequence alignment of UspC from *Mtb* with UspC homologues from other mycobacteria.

The sequence alignment was generated using Clustal Omega (www.ebi.ac.uk/Tools/msa/clustalo/) and ESPript version 3 [**1**]. Numbering corresponds to the sequence of *Mtb* UspC. Identical residues are indicated by a red background, and conserved residues are indicated by red characters. Secondary structure elements of *Mtb* UspC are shown above the sequences.

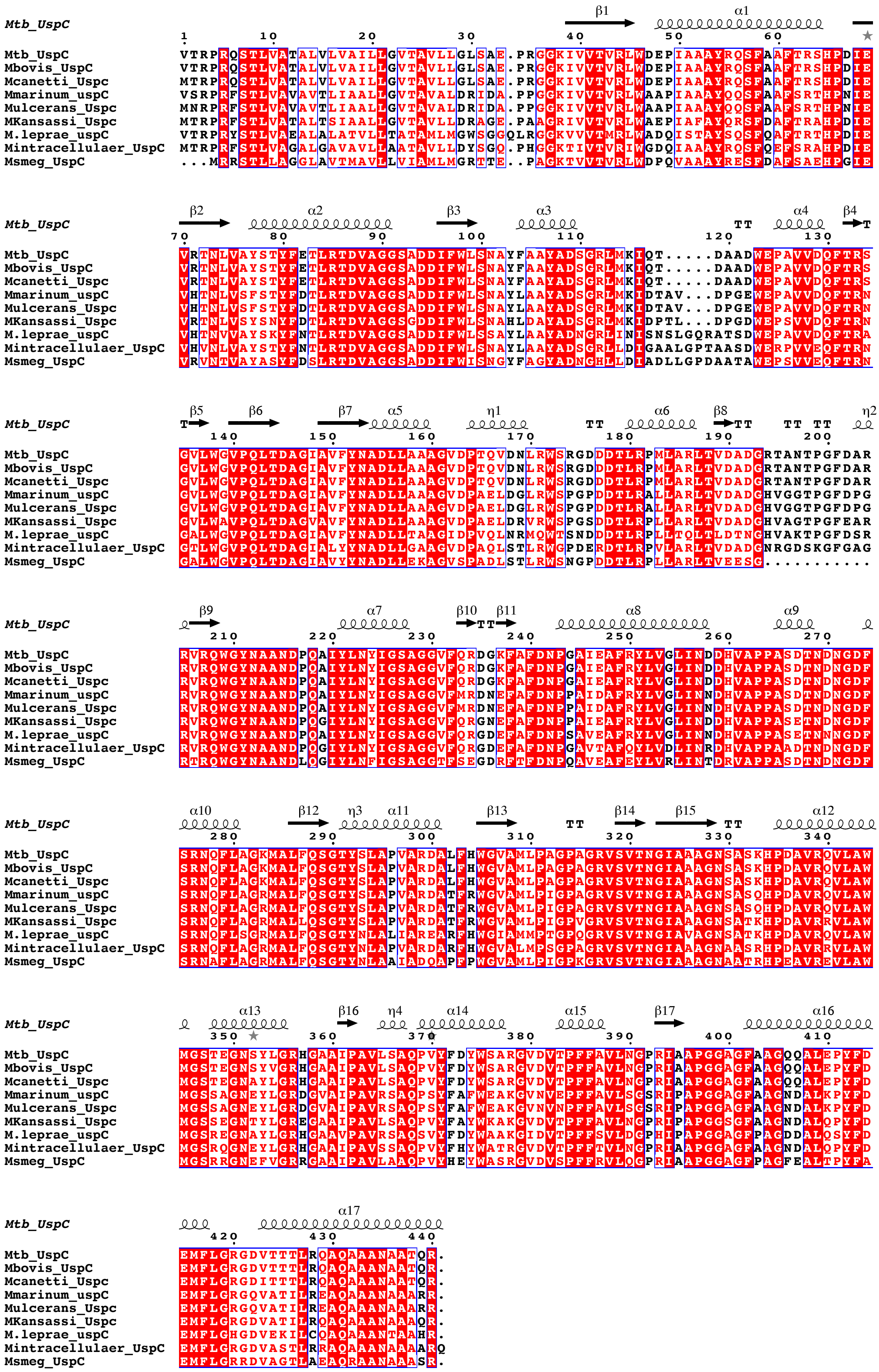

Figure S2. SDS-PAGE analysis of the purification of UspC$_{Nt}$ from *E. coli* cell extracts. a) Elution of His$_6$-tagged UspC$_{Nt}$ from a Ni-NTA column. Mr = molecular weight markers in kDa, WC = cell lysate, IS = insoluble fraction, FT = column flow through, numbers of 0 to 250 refer to the imidazole concentration in the elution buffer (units of mM). b) QHP anion exchange chromatography of UspC$_{Nt}$ following the Ni-NTA purification step. FT = flow through, numbers across the top indicate NaCl concentration in the elution buffer (units of mM). See Materials and methods for buffer compositions.

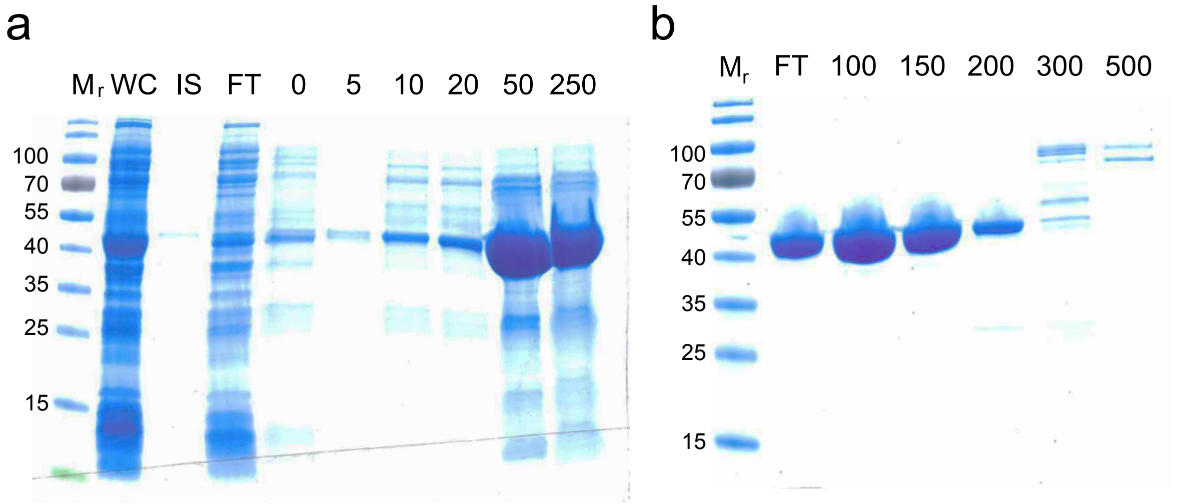

Figure S3. Experimental electron density map after SAD phasing and multi-crystal averaging. The structure of UspC$_{Nt}$ was determined by single-wavelength anomalous diffraction of an iodine derivative, and an interpretable map was obtained after solvent flattening and averaging density across two crystal forms. The nominal resolution of the map is 2.6 Å, and it is contoured at 1.2σ above the mean density.

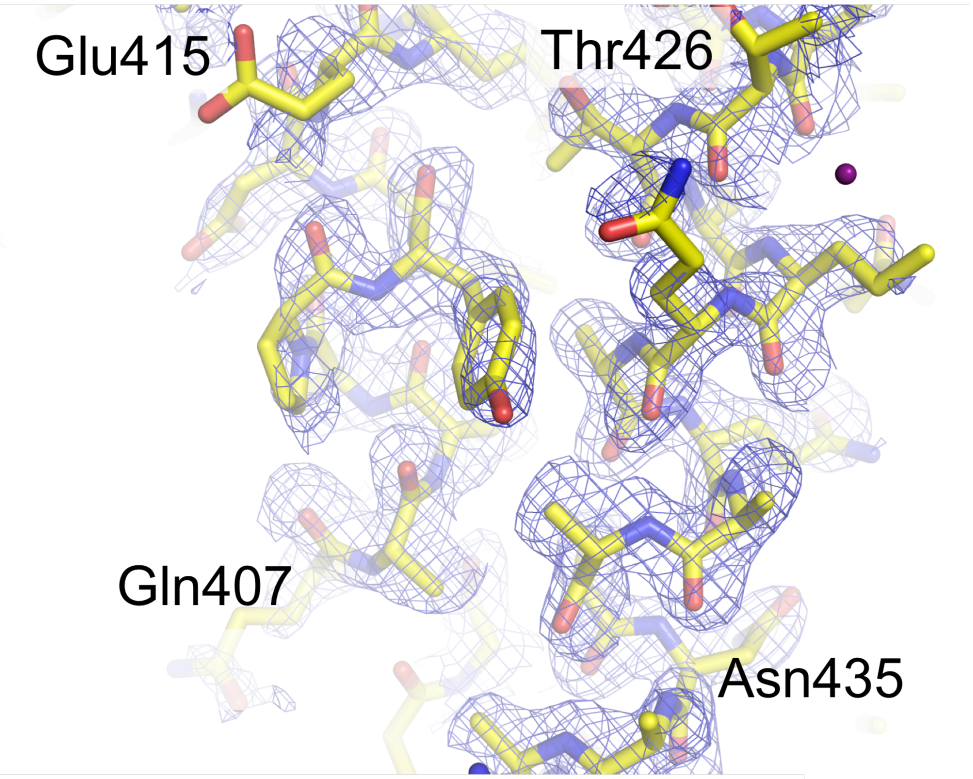

Figure S4. Comparison of the open and closed forms of the GacH receptor of *Streptomyces glaucescens*. a) Ribbon diagram of the *apo* (grey) and maltotetraose-bound GacH (pale green) (PBD entries 3K01, 3K00, [**2**]). b, c) molecular surface representation of the same structures, coloured according to panel *a*. Maltotetraose is shown as a stick model.

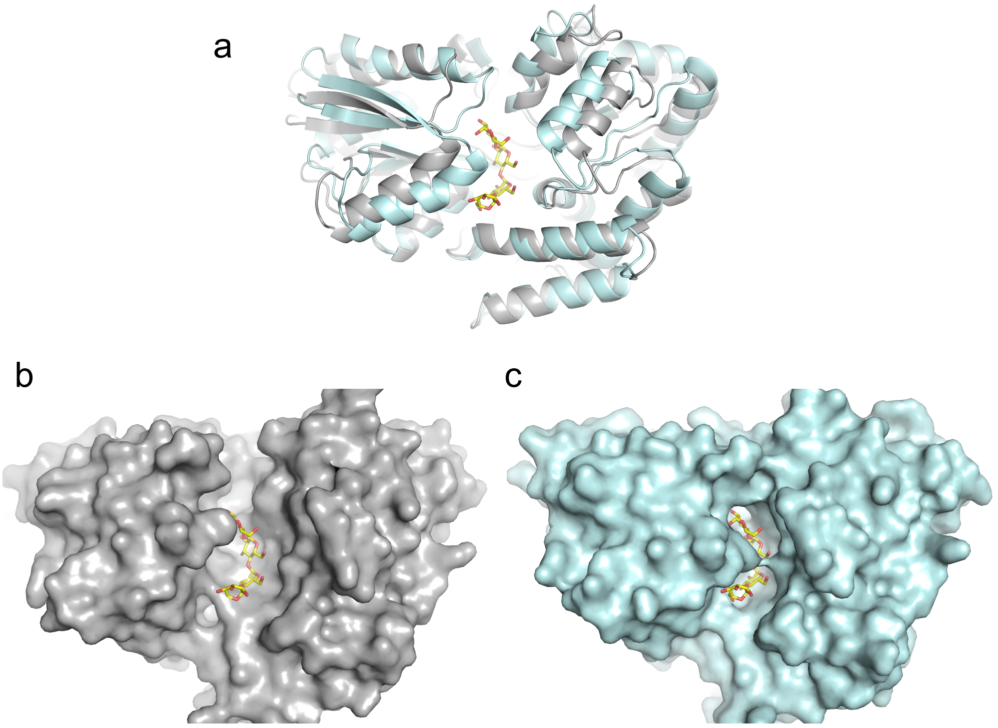

Figure S5. Size exclusion chromatography of purified UspC$_{Nt}$. Column resin was Superdex S200, buffer composition: 20 mM Hepes, pH 7.6, 100 mM NaCl, 10% v/v glycerol. Sizing markers were alcohol dehydrogenase (150 kDa), bovine serum albumin (66 kDa) and carbonic anhydrase (29 kDa). The sequence-derived molecular weight of UspC$_{Nt}$ is 44 kDa. Elution was monitored by recording absorbance at 280 nm.

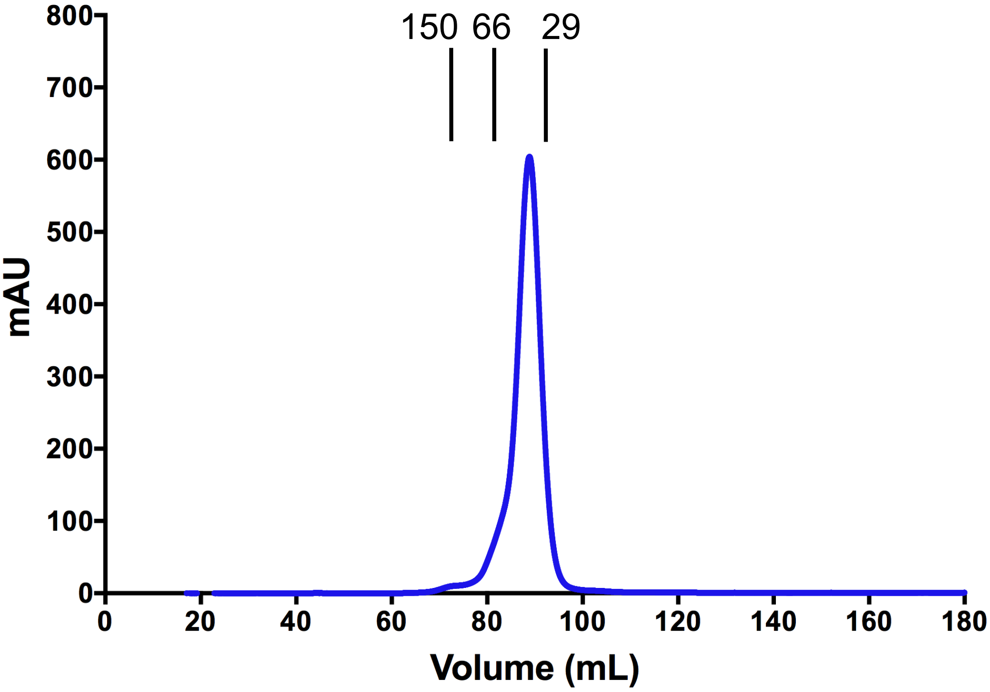

Figure S6. Comparison of $UspC_{Nt}$ with structures of ABC-transporter solute-binding proteins. a) Superposition of $UspC_{Nt}$ (blue, orange) with the *E. coli* MBP-maltose transporter (PDB entry 3PUW, [**3**]). Chain E (solute binding unit) of the maltose transporter is shown as a pale green ribbon, while the other transporter polypeptides are shown in grey. b) Superposition of $UspC_{Nt}$ with the structure of UgpB (pale blue, PDB entry 4MFI, [**4**]).

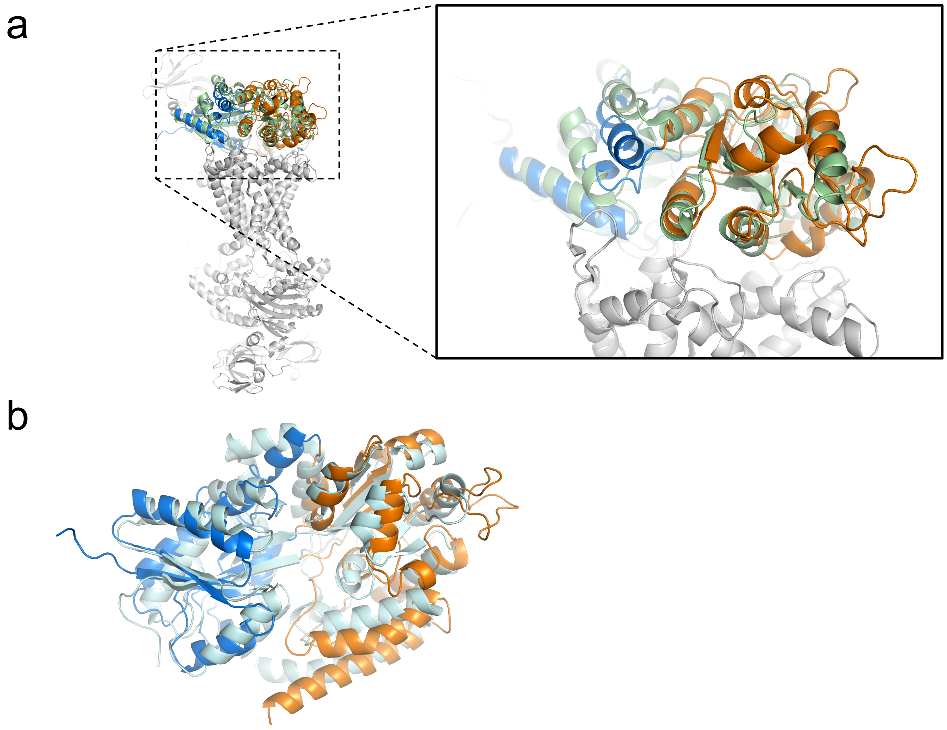

Figure S7. Side-by-side comparison of electrostatic surfaces of UspC, *Mtb* UgpB and *Streptomyces glaucescens* GacH. Molecular surfaces were calculated using CCP4MG [**5**] and are coloured according to surface charge (red = negative, blue = positive). a) UspCNt; b) UgpB (4MFI, [**4**]); c) maltotetraose-bound *S. glaucescens* GacH (3K00, [**2**])

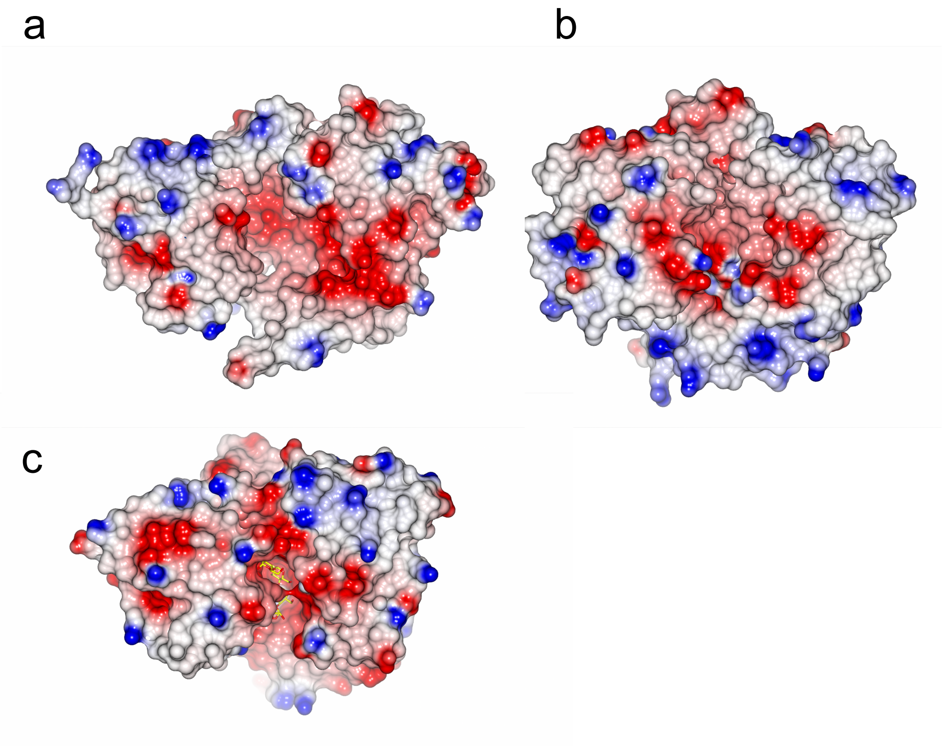

Figure S8. Thermal shift assay of additional non-binding carbohydrates to UspC. a) Bar graph illustrating shifts of T_m for a series of carbohydrates. Data shown are from three independent repeats. $UspC_{Nt}$ mutants carrying a single alanine substitution at Asp145, Gln218 or Tyr292 are labelled as 145, 218 or 292, respectively. Chitobiose (also included in Figure 3a is included for comparison).

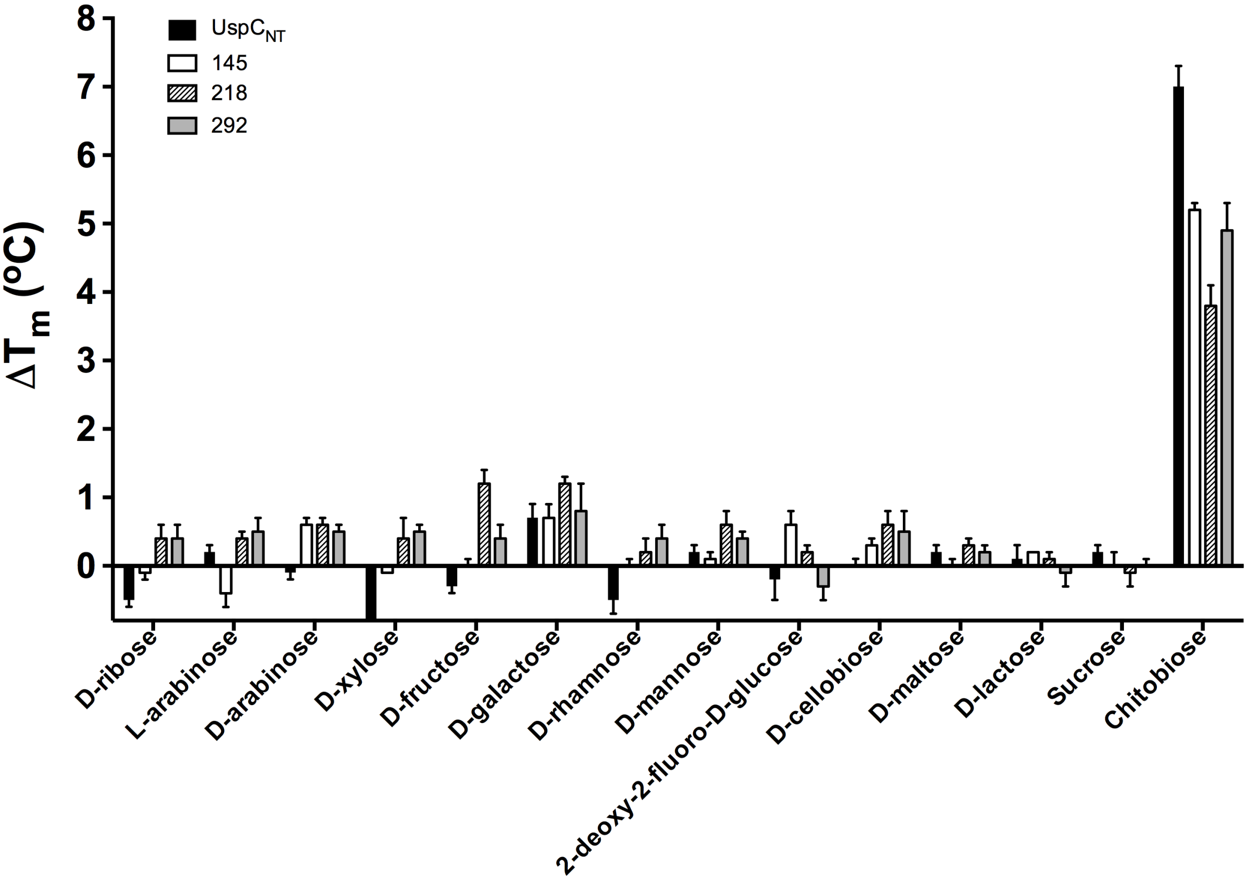

Supplement: Supplementary Information - combined [file rsob160105supp1.pdf]
